# Comparative Proteomic Profiling of Human Bile Reveals SSP411 as a Novel Biomarker of Cholangiocarcinoma

**DOI:** 10.1371/journal.pone.0047476

**Published:** 2012-10-31

**Authors:** Jian Shen, Weizhi Wang, Jindao Wu, Bing Feng, Wen Chen, Meng Wang, Jincao Tang, Fuqiang Wang, Feng Cheng, Liyong Pu, Qiyun Tang, Xuehao Wang, Xiangcheng Li

**Affiliations:** 1 Key Laboratory of Living Donor Liver Transplantation, Ministry of Public Health, Department of Liver Transplantation Center, The First Affiliated Hospital of Nanjing Medical University, Nanjing, China; 2 Department of General Surgery, The Second Affiliated Hospital of Nanjing Medical University, Nanjing, Jiangsu, China; 3 Department of Thoracic and Cardiovascular Surgery, Nanjing First Hospital Affiliated to Nanjing Medical University, Nanjing, China; 4 Laboratory of Reproductive Medicine, Department of Histology and Embryology, Nanjing Medical University, Nanjing, China; 5 Department of Gastroenterology, The First Affiliated Hospital of Nanjing Medical University, Nanjing, China; Queensland Institute of Medical Research, Australia

## Abstract

**Background:**

Cholangiocarcinoma (CC) is an intractable cancer, arising from biliary epithelial cells, which has a poor prognosis and is increasing in incidence. Early diagnosis of CC is essential as surgical resection remains the only effective therapy. The purpose of this study was to identify improved biomarkers to facilitate early diagnosis and prognostication in CC.

**Methods:**

A comparative expression profile of human bile samples from patients with cholangitis and CC was constructed using a classic 2D/MS/MS strategy and the expression of selected proteins was confirmed by Western blotting. Immunohistochemistry was performed to determine the expression levels of selected candidate biomarkers in CC and matched normal tissues. Finally, spermatogenesis associated 20 (SSP411; also named SPATA20) was quantified in serum samples using an ELISA.

**Results:**

We identified 97 differentially expressed protein spots, corresponding to 49 different genes, of which 38 were upregulated in bile from CC patients. Western blotting confirmed that phosphoglycerate mutase 1 (brain) (PGAM-1), protein disulfide isomerase family A, member 3 (PDIA3), heat shock 60 kDa protein 1 (chaperonin) (HSPD1) and SSP411 were significantly upregulated in individual bile samples from CC patients. Immunohistochemistry demonstrated these proteins were also overexpressed in CC, relative to normal tissues. SSP411 displayed value as a potential serum diagnostic biomarker for CC, with a sensitivity of 90.0% and specificity of 83.3% at a cutoff value of 0.63.

**Conclusions:**

We successfully constructed a proteomic profile of CC bile proteins, providing a valuable pool novel of candidate biomarkers. SSP411 has potential as a biomarker for the diagnosis of CC.

## Introduction

Cholangiocarcinoma (CC) is a primary malignancy which originates from bile duct epithelial cells. CC approximates 10 to 25% of all liver cancers and the incidence of this disease has increased over the last three decades [1], [2]. CC is a slow-growing but highly metastatic tumor, which is often detected at an unresectable stage; therefore, most patients have a poor prognosis with a median survival of 6–12 months [3]. CC is insensitive to chemotherapy, immunotherapy, radiotherapy and other adjuvant treatments, and curative surgical resection is currently the only effective therapy, with an overall 5-year survival rate of 40% [4], [5]. However, more than a third of patients with CC are unsuitable candidates for curative resection, as the disease is usually detected at an advanced stage. Hence, new methods of early diagnosis are urgently required in order to improve the treatment and prognosis of CC patients.

Currently, the clinical diagnosis of CC relies on computed tomography (CT) or B type ultrasonography examinations which have a poor sensitivity, especially for the detection of small lesions with a hilar localization. In addition, brush cytology via endoscopy has a sensitivity of 50% for the early diagnosis of CC, which is attributed to the high desmoplastic nature of this disease [6]. The serum biomarker CA 19-9 is commonly used for the diagnosis of CC; however, CA 19-9 has low sensitivity of 50–60% and specificity of 80% [3]. Therefore, improved fluid-based biomarkers are urgently required to enable the early diagnosis of CC, and additional insight on the pathogenesis of this disease is critical in order to identify new potential therapeutic strategies.

Proteomics is the most commonly used technology for the identification of disease-specific biomarkers. The protein expression profiles of normal cells undergo distinct changes during malignant transformation, which may potentially provide appropriate biomarkers [7]. In CC, the bile drainage proteins directly secreted/shed by tumor cells may accumulate to higher concentrations in bile than serum, and may therefore be easier to identify in bile [8], [9]. Although a few studies have attempted to perform large-scale identification of differently expressed bile proteins in CC [8], [10]–[15], most of this research has focused on improvements in proteomic methodologies, or extension of the human bile proteomic profile in single or manipulus patients. Consequently, we performed a comparative proteomic analysis of human bile obtained from patients with CC and patients with benign disease, in order to potentially identify novel biomarkers for CC using a standard two dimensional gel electrophoresis (2-DE) strategy.

## Materials and Methods

### Ethical approval

All samples and clinical information were collected at the Liver Transplantation Center of the 1^st^ Affiliated Hospital of Nanjing Medical University, and all patients provided written informed consent. The study was approved by the Ethics Committee of Nanjing Medical University with an IEC number of 2011-SRFA-012. The detailed patient characteristics are presented in Table 1.

**Table 1:** Clinical characteristics of the patients included in this study.

	Characteristics	No. of individuals
**CC group (35)**		
	Gender(male/female)	20/15
	Age (mean ± SD)	60.7±10.6 yr
	CC type (hilar/-perihilar IHC)	17/8
	Histopathology (well/moderately/poorly)	10/8/9
	Lymph node metastasis (P/N)	15/12
	Nerve invasion (P/N)	23/4
	Sample source (bile/serum)	19/30
**Benign group (13)**		
	Gender(male/female)	7/6
	Age (mean ± SD)	46.5±12 yr
	Sample source (bile/serum)	10/13
**Normal group (23)**		
	Gender(male/female)	13/10
	Age (mean ± SD)	48.3±13.7 yr
	Sample source (bile/serum)	0/23
**HCC group (24)**		
	Gender(male/female)	17/7
	Age (mean ± SD)	52.1±13.9 yr
	Sample source (bile/serum)	0/24
**Liver cirrhosis (10)**		
	Gender(male/female)	7/3
	Age (mean ± SD)	42.9±10.9 yr
	Sample source (bile/serum)	0/10

CC: patients with cholangiocarcinoma (including eight patients that received non-surgical treatment); IHC: intrahepatic cholangiocarcinoma; HCC: patients with hepatocellular carcinoma; benign group: patients with cholangitis; Normal: healthy people. P: positive, N: negative.

### Sample collection and preparation

The blood samples were centrifuged for 3,000 rpm/min at 4°C, and the serum was collected and frozen at −80°C until analysis. Fresh tissues were procured at the time of surgery and divided into two parts: one part was washed with saline to remove blood and bile and then snap-frozen in liquid nitrogen, the other part was formalin-fixed and paraffin-embedded for HE staining or immunohistochemistry. All bile samples were collected from the gallbladder or dilated bile duct before resection during surgery under sterile conditions; a protease inhibitor (Pierce Biotechnology, Rockford, IL, USA) was added and samples were stored at −80°C until processing. The bile proteins were enriched as previously described [8].

### Depletion of the high-abundance proteins in bile

Depletion of the high-abundance proteins was performed using Multiple Affinity Removal System (MARS) columns (Agilent, Palo Alto, CA, USA), which are designed to deplete 14 abundant proteins, according to the manufacturer's protocol. The protein concentrations of the processed bile samples were determined using the Bradford method (Beyotime, China) using BSA as a standard.

### Two-dimensional electrophoresis and MALDI-TOF/TOF

Bile samples from 15 CC patients and 10 cholangitis patients were used for the 2-DE experiment. In the benign group, six individual samples were pooled in groups of three to create two samples, and a third pooled sample contained four individual samples. In the tumor group, the individual samples were mixed in groups of five to create three pooled samples. In brief, 120 µg protein samples were separated by 2D PAGE and visualized using silver staining. ImageMasterTM 2-D Platinum Software (Amersham Bioscience, CA, USA) was used for comparative analyses (Student's *t*-tests; *P* values<0.05 were considered statistically significant) and the differentially expressed protein spots were excised and identified as previously described [16], [17]. Briefly, the protein spots were dehydrated in acetonitrile (ACN), and dried at room temperature. Spots were reduced using 10 mM DTT/25 mM NH4HCO3 at 56°C for 1 h and subsequently alkylated in situ with 55 mM iodoacetamide/25 mM NH4HCO3 in the dark at room temperature for 45 min. Gel fragments were thoroughly washed with 25 mM NH4HCO3, 50% ACN, and 100% ACN and dried in a SpeedVac. Dried gel fragments were re-swollen with 2–3 µL trypsin solution (Promega, Madison, WI, USA) (10 ng/µL in 25 mM NH4HCO3) at 4°C for 30 min. Excess liquid was discarded and the gel plugs incubated at 37°C for 12 h. Trifluoroacetic acid (TFA) at a final concentration of 0.1% was added to stop the digestive reaction.

The digests were immediately spotted onto 600-µm AnchorChips (Bruker Daltonics, Bremen, Germany). Spotting was achieved by pipetting 1 µL of the analyte onto the MALDI target plate in duplicate and subsequently adding 0.05 µL of 2 mg/mL α-HCCA in 0.1% TFA/33% acetonitrile containing 2 mM (NH4)3PO4. Bruker peptide calibration mixture (Bruker Daltonics) was also spotted for external calibration. All samples were air-dried at room temperature and 0.1% TFA was used for on-target washing. All samples were analyzed on a time-of-flight Ultraflex II mass spectrometer (Bruker Daltonics) set to the positive-ion reflectron mode.

Each acquired mass spectrum (m/z range, 700–4000; resolution, 15000–20000) was processed using the FlexAnalysis v2.4 and Biotools 3.0 (Bruker Daltonics) software packages with the following specifications: peak detection algorithm, Sort Neaten Assign and Place (SNAP); S/N threshold, 3; and quality factor threshold, 50. Trypsic autodigestion ion picks (842.51, 1045.56, 2211.10, and 2225.12 Da) were used as internal standards to validate the external calibration procedure. Matrix and/or autoproteolytic trypsin fragments were removed. The masses of the peptides obtained were cross-referenced with the NCBI human database with the use of Mascot (v2.1.03) in an automated mode that used the following search parameters: a significant protein score at *P*<0.05; minimum mass accuracy 100 ppm; trypsin as the enzyme; one missed cleavage sites allowed; cysteine carbamidomethylation, acrylamide modified cysteine, methionine oxidation and similarity of pI, and the relative molecular mass specified, with the minimum sequence coverage at 15%.

Protein identification was confirmed by sequence information automatically obtained from the MS/MS analysis. Acquired MS/MS spectra were also processed using the software FlexAnalysisTM 2.4 using a SNAP method set at a signal-to-noise ratio threshold of 3.0. The MS/MS spectra were automatically searched in the NCBI human database by Mascot (v2.4). Search parameters for MS/MS data were set to 100 ppm for the precursor ion and 0.3 Da for the fragment ions. Cleavage specificity and covalent modifications were considered, as described above. The score was higher than the minimum significant individual ion score (P<0.05). All significant MS/MS identifications by Mascot were manually verified for spectral quality and matching y and b ion series. When multiple entries corresponded to slightly different sequences, only the database entry that exhibited the highest number of matching peptides was included.

### Western blot analysis

Pooled bile and tissue proteins (40 µg) or crude bile (2 µl) from individual patients were resolved on SDS-PAGE gels, transferred onto PVDF membranes (Millipore, Bedford, MA, USA) and incubated overnight with primary antibodies against PGAM1 (1∶1000; Abnova, Taibei, Jhouzih St, Taiwan), HSPD1 (1∶1,000; Abcam, Cambridge, MA, USA), SSP411 (1∶1,000; Abgent, San Diego, CA, USA), APOM (1∶100; Santa Cruz Biotechnology, Santa Cruz, CA, USA), Pdia3 (1∶500; Abcam) and GAPDH (1∶5,000; Abcam). Ponceau S staining was used as a loading control after membrane transfer [18], [19] and GAPDH was used as an internal control. The membranes were incubated with horseradish peroxidase (HRP)-conjugated secondary antibody (1∶4,000; Beijing ZhongShan Biotechnology, Beijing, China) for 1 h, the bands were visualized using an ECL detection kit (Pierce-Thermo Scientific, Rockford, IL, USA), following the manufacturer's instructions and the relative signal intensity of each target protein was quantified using Quantity One software (Bio-Rad, Hercules, CA, USA).

### Immunohistochemistry

Serial 4-µm sections of each specimen were deparaffinised and rehydrated before antigen retrieval was performed by microwaving the slides in 10 mM citric acid buffer (pH 7.0). After elimination of endogenous peroxidase activity, the specimens were blocked with blocking serum (Santa Cruz Biotechnology) and incubated with primary anti-PGAM-1, anti-SSP411, anti-HSPD1 (all 1∶200) or anti PDIA3 (1∶1000) antibodies at 4°C overnight. Negative controls were incubated in a solution devoid of primary antibody. The sections were incubated with HRP-conjugated secondary antibody for 1 h, staining was visualized using diaminobenzadine and images were obtained using bright-field microscopy (Axioskop 2 plus; ZEISS, Germany).

### Quantification of SSP411 serum levels

Serum samples from 30 CC patients, 13 benign hepatobiliary disease patients and 23 normal individuals were used for the ELISA analysis. The serum samples were diluted 1∶1000, directly adsorbed to 96-well plates overnight at 4°C, blocked with 5% non-fat milk powder and incubated with SSP411 primary antibody (1∶2,000) for 1 h at 37°C. The plate was incubated with HRP-conjugated secondary antibody (1∶3,000; Golden Bridge, China), visualized using TMB solution (Beyotime, China) and color intensity was measured at a wavelength of 420 nm (using 630 nm as the background control). MedCalc software (MedCalc, Belgium) was used for statistical analyses of the receiver operator characteristic (ROC) curves and areas under the curve (AUC).

## Results

### Sample preparation optimization and construction of the comparative human bile proteomic profile

**Figure 1:**
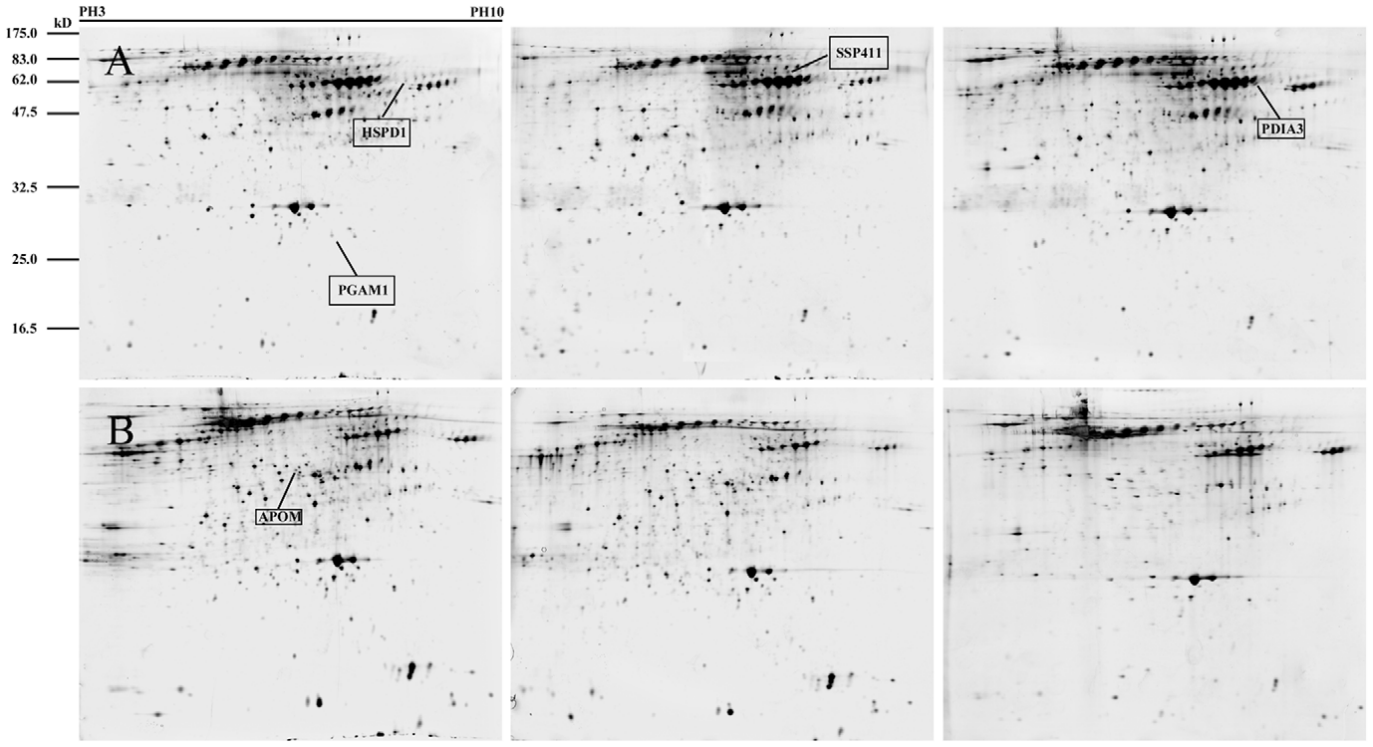
2-DE gels of pooled human bile samples. (A, B) 2-DE maps of pooled bile samples from cholangiocarcinoma patients (A; n = 5 each) and pooled samples from cholangitis patients (B; n = 3 or 4 each). The proteins were separated by 2D-PAGE and visualized by silver staining. The differentially expressed proteins selected as potential biomarkers are marked.

Two-dimensional electrophoresis was performed on bile samples from 15 CC and 10 cholangitis patients (pooled as described in the Material and Methods) over a pH range of 3–10 (Figure 1). Analysis of the 2-DE gels revealed 109 spots were differently expressed in the pooled CC and cholangitis bile samples (*P*<0.05). In total, 97 spots corresponding to 49 genes were successfully identified via MALDI-TOF/TOF. Additionally, a number of proteins yielded more than two spots in the profiles (Table S1). Among the 97 proteins, 61 proteins were present in a higher abundance in bile from CC patients compared to the cholangitis group. Bioinformatic analysis of the 49genes by the BioGPS database (http://biogps.org/#goto=welcome) revealed that eight genes were uniquely expressed by the liver, while the other 14 were highly expressed in the liver or fetal liver (Figure S1). These results suggest that the successfully identified differentially expressed proteins were derived from bile.

### Verification of candidate biomarkers in the pooled and individual bile samples

**Figure 2:**
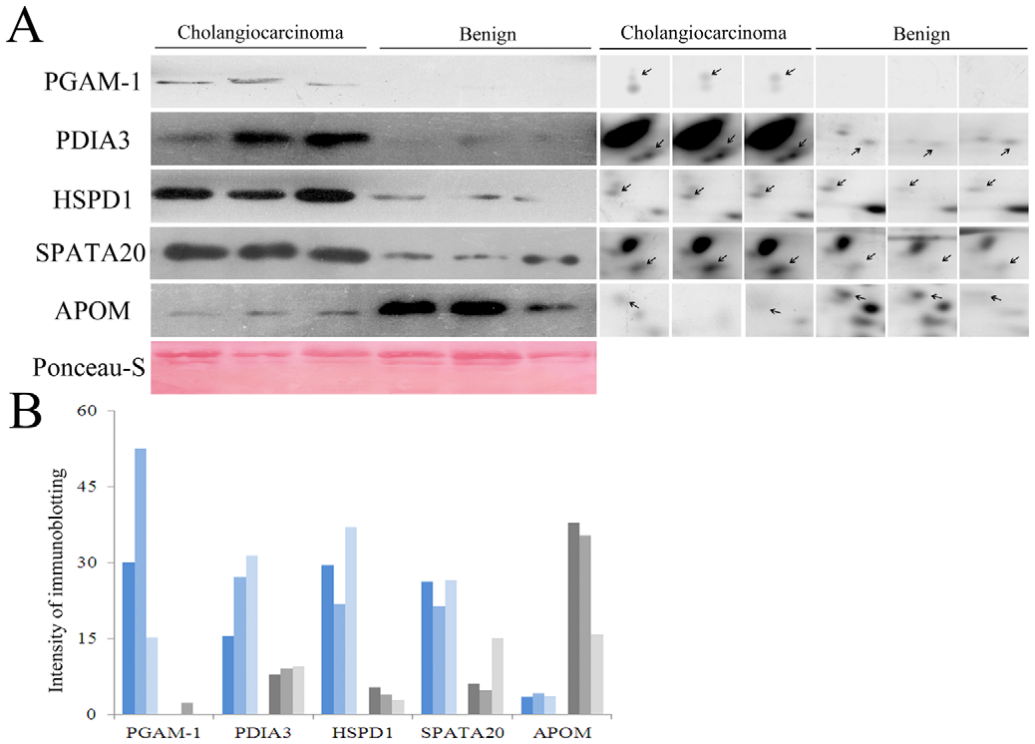
Validation of the 2-DE results by Western blotting. (A) Left panel: Western blotting analysis of aliquots of pooled bile proteins from patients with CC or cholangitis. Right panel: the corresponding spots, with the same molecular weights, in the 2-DE gels. The Ponceau-S-stained blot (below) was used the internal control. (B) Quantification of protein expression in pooled bile proteins. The bars represent the signal intensity of the immunobands and were consistent with the 2-DE results.

To verify the proteomic analysis, five proteins were randomly selected for immunoblotting analysis: PGAM-1, PDIA3, HSPD1 and SSP411 which were upregulated in CC bile and APOM which was downregulated in CC bile. The proteins migrated at the expected molecular weights (Figure 1) and Western blotting revealed that the expression levels of these proteins in the pooled CC and cholangitis bile samples were essentially consistent with the 2D-PAGE results (Figure 2). Additionally, Western blotting of SSP411, PGAM-1, PDIA3 and HSPD1 in individual bile samples provided identical results to the pooled bile samples.

**Figure 3:**
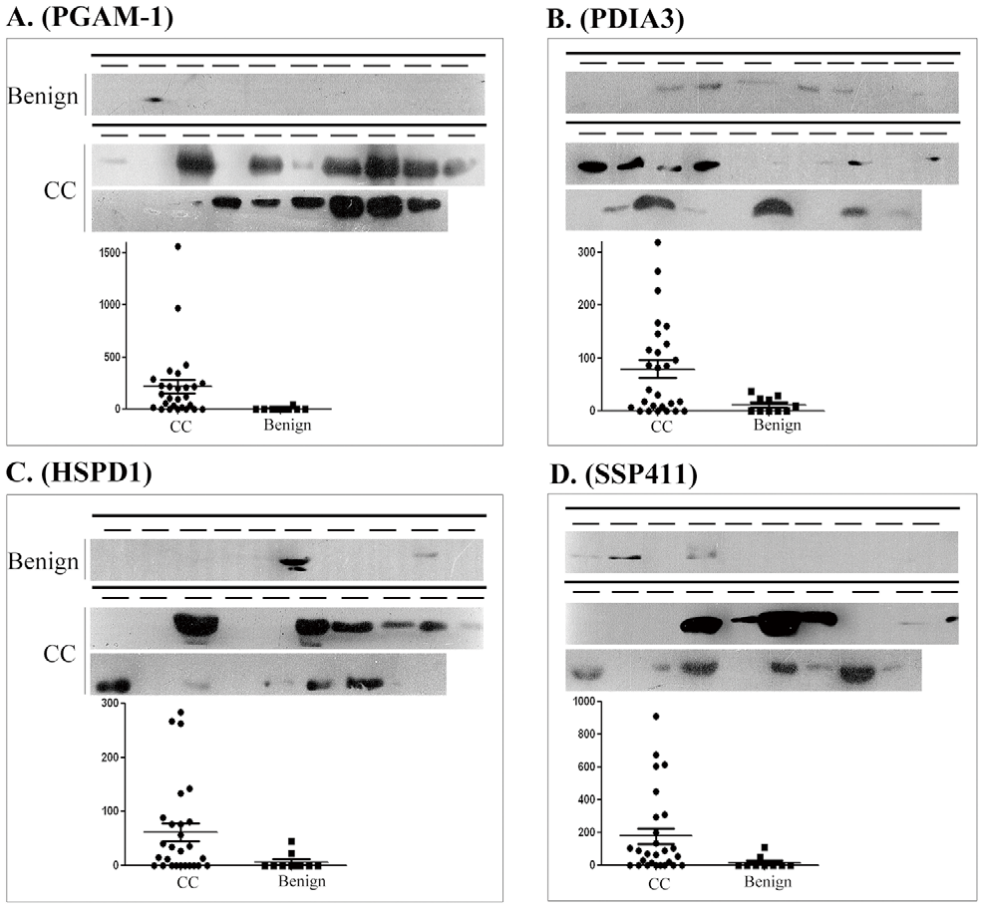
Western blot validation of four candidate cholangiocarcinoma biomarkers in individual bile samples. Western blotting (top) and quantification (bottom) of candidate biomarker expression in equal volumes of individual bile samples from 10 cholangitis patients (benign) and 19 cholangiocarcinoma (CC) patients. (A) PGAM-1; (B) PDIA3; (C) HSPD1 (D) and SSP411.

In Western blots of crude bile (2 µl), PGAM1 and SSP411 were barely detectable in the benign group but were expressed at high levels in the CC group (Figure 3A and D). PDIA3 and HSPD1 (Figure 3B and C) could be detected in several cholelithiasis patients; however, the average expression levels of PDIA3 and HSPD1 in crude bile from CC patients was significantly higher.

### Validation of PGAM-1, HSPD1, PDIA3 and SSP411 expression in surgical tissues by immunoblotting and immunohistochemical analysis

**Figure 4:**
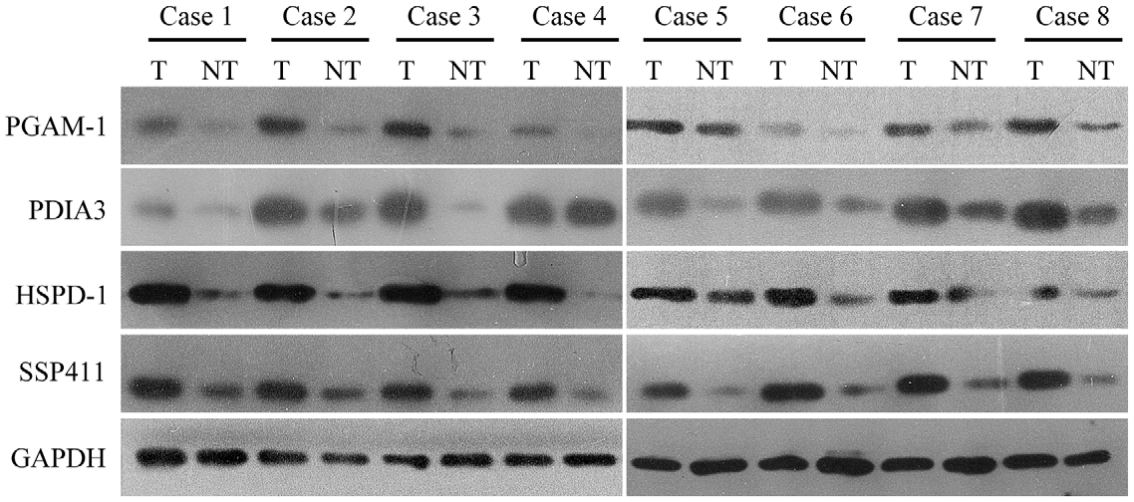
Western blot validation of candidate biomarker expression in paired cholangiocarcinoma and normal surgical tissue samples. The candidate biomarkers PGAM-1, PDIA3, HSPD1, and SSP11 were expressed at higher levels in cancerous tissues (T) compared to paired normal tissues (NT). GAPDH was used as loading control.

Western blotting was used to quantify the expression of PGAM-1, HSPD1, PDIA3 and SSP411 (which were all upregulated in bile from CC patients) in eight pairs of CC and adjacent non-tumor bile duct tissues. As shown in Figure 4, PGAM-1, HSPD1, PDIA3 and SSP411 were overexpressed in tumor tissues compared to the matched non-tumor tissues.

**Figure 5:**
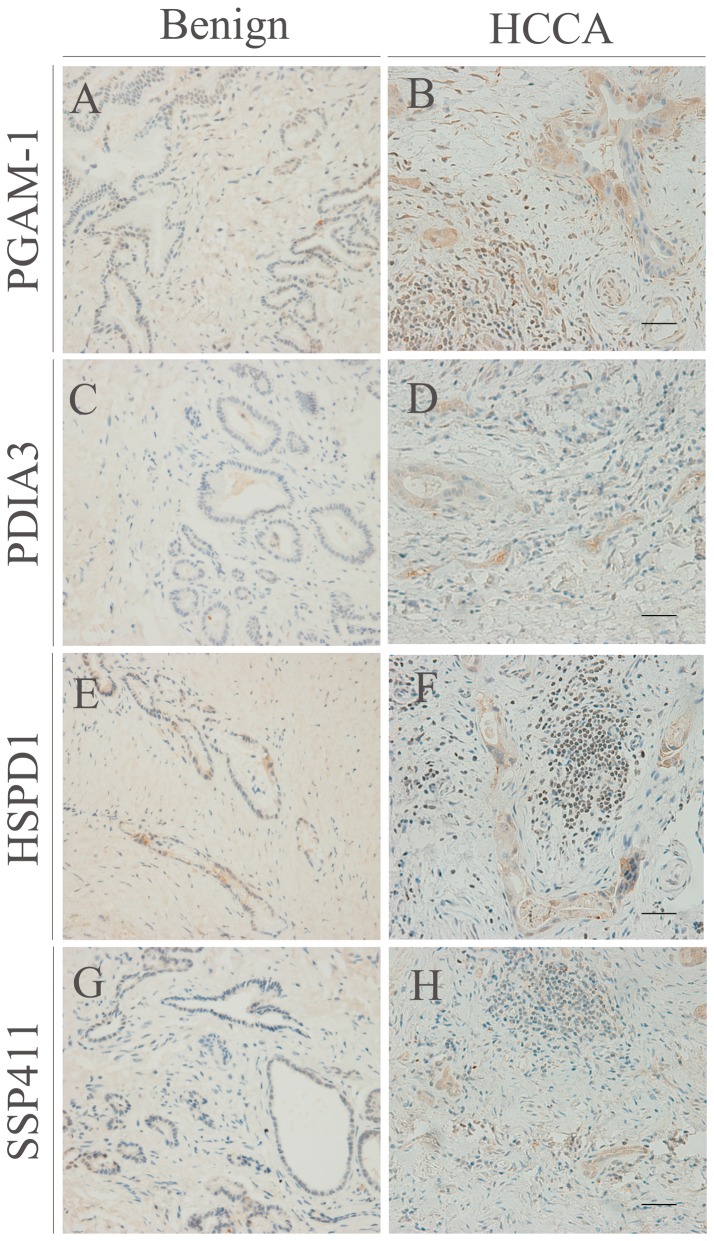
Immunohistochemical analysis of PGAM-1, PDIA3, HSPD1 and SSP411 in hilar cholangiocarcinoma (HCCA). Differences in the expression of PGAM-1 (A, B), PDIA3 (C, D), HSPD1 (E, F), SSP411 (G, H) in cancerous (right) versus normal tissue specimens (left). Immunohistochemical staining profiles in intrahepatic cholangiocarcinoma (IHC) are shown in Figure S2. Bar = 20 µm.

Immunohistochemical analysis was performed to characterize the distribution of PGAM-1, HSPD1, PDIA3 and SSP411 in surgical tumor tissues. All four proteins were upregulated in CC (Figure 5, right panel) compared to the non-tumor tissues came from patients with choledocholithiasis (Figure 5, left panel). Intense PGAM-1, HSPD1, PDIA3 and SSP411 cytoplasmic immunoreactivity was observed in both hilar cholangiocarcinoma (Figure 5) and intrahepatic cholangiocarcinoma (Figure S2). The lumen of the cancer nests also demonstrated various intensities of immunostaining, suggesting that the proteins can be secreted extracellularly. The immunohistochemical analysis provided further confirmation that PGAM-1, HSPD1, PDIA3 and SSP411 were overexpressed in CC, suggesting that these proteins may provide potential biomarkers for CC.

### Evaluation of the serum levels of SSP411 by ELISA as a diagnostic test

**Figure 6:**
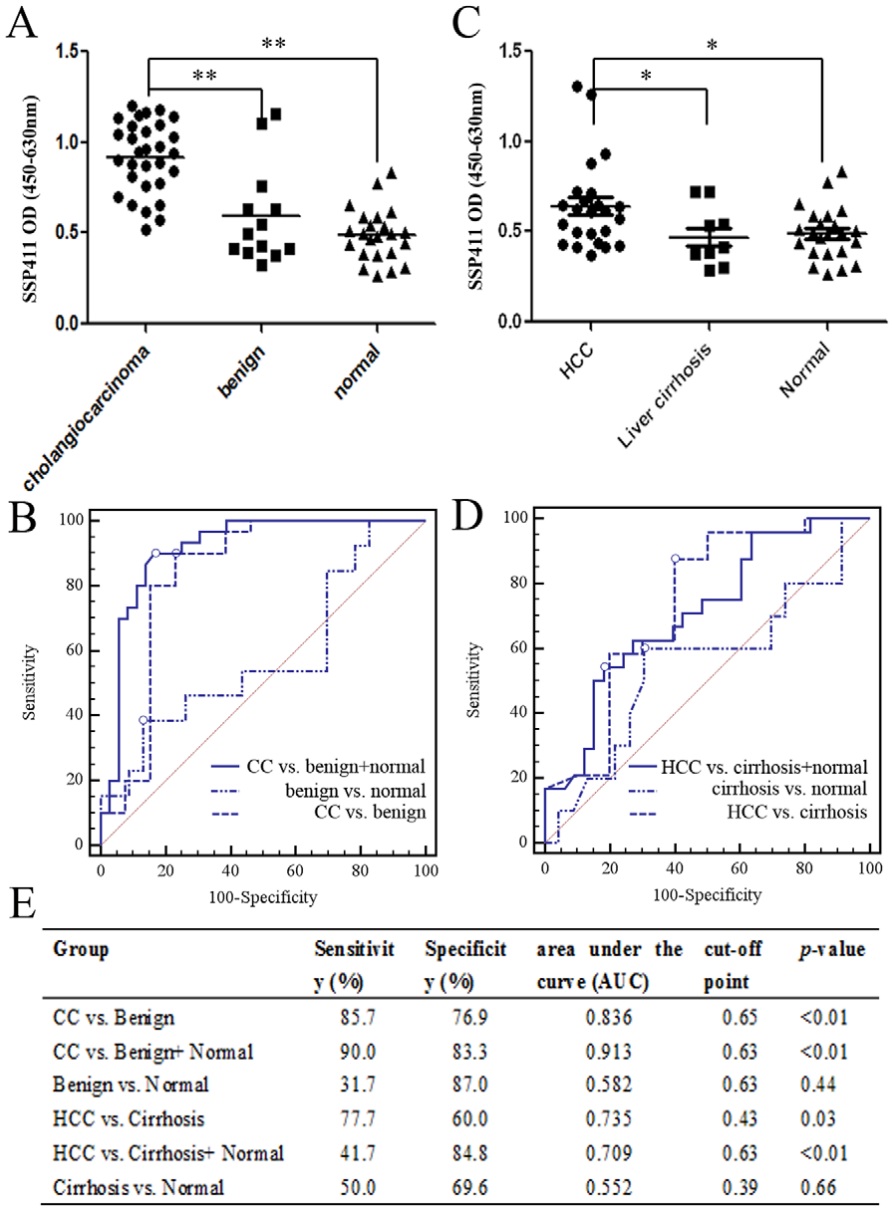
Validation of the diagnostic value of SSP411 in serum samples using an ELISA. (A). Distribution of the serum OD value in cholangiocarcinoma (CC) patients, patients with benign disease and healthy individuals. (B). Receiver operating characteristic (ROC) analysis for the optimal cut-off point for discrimination between between different groups (CC vs. benign; CC vs. benign+normal; benign vs. normal). (C). Distribution of the serum OD values in hepatocellular carcinoma (HCC) patients, patients with liver cirrhosis and healthy individuals. (D). ROC analysis of SSP411 for HCC. (E). ROC analysis results between different CC and HCC groups.

As bile can only be collected during surgery, bile biomarkers are not suitable as a pre-surgical diagnostic tool. Therefore, we examined the serum levels of one potential biomarker. BioGPS analysis indicated SSP411 is a testis-enriched gene which is not expressed in normal liver (Figure S1C). SSP411 has not previously been associated with other cancers. The diagnostic value of SSP411 as a novel candidate serum diagnostic biomarker for CC was tested using an ELISA assay and receiver operating characteristic (ROC) analysis. Consisted with the proteomic results, CC patients had significantly higher serum levels of SSP411 (mean OD value ± SD of 0.92±0.20) than individuals from the normal (0.49±0.15; *P*<0.01) and benign groups (0.59±0.27; *P*<0.01, Figure 6A).The ROC area under the curve (AUC) for SSP411 was 0.913 and the cut-off point was 0.63, with a sensitivity of 90% and a specificity of 83.3% to distinguish CC from benign disease and normal controls. The AUC was 0.836 and the cut-off point was 0.65 (sensitivity = 85.7; specificity = 76.9) when the CC was compare with the benign group (Figure 6E). Similar to CC, the SSP411 level in HCC patients (0.64±0.24) were higher than in the liver cirrhosis (0.47±0.16; *p<0.05*) and normal groups (0.49±0.15; *p<0.05*; Figure 6C). However, the diagnostic efficiency of SSP411 for HCC was significantly lower than for CC (Figure 6D). The sensitivity (HCC vs. cirrhosis, 77.7%; HCC vs. cirrhosis+ normal, 41.7%) and specificity (HCC vs. Cirrhosis, 60.0%; HCC vs. Cirrhosis+ Normal, 84.8%) of SSP411 for the diagnosis of HCC were not satisfactory (Figure 6, E).

## Discussion

Cholangiocarcinoma (CC) is the second most common primary hepatic malignancy of the biliary-duct system. The typical age of CC is the seventh decade of life, with a slightly higher incidence in men [20]. Our study found an average age of 60.7±10.6 yr and male patients were also more likely to be affected than female patients with a ratio of 1.3. Given the poor prognosis of CC, mortality and incidence rates are virtually similar. CC incidence rates vary markedly worldwide, which presumably reflects differences in local risk factors and genetic susceptibility. There are a number of established risk factors underlying CC carcinogenesis, such as primary sclerosing cholangitis (PSC), infestation with liver flukes, toxic, biliary-tract disorders, hepatolithiasis, choledocholithiasis and cholangitis, amongst others. However, most patients that present with CC do not have identifiable risk factors [21].

PSC is the most common predisposing factor for CC in the Western countries. This is an autoimmune disease that causes structuring of the biliary tree. Approximately 40% of patients with PSC will eventually develop CC, but this is not correlated with the duration of PSC [22], [23]. The possible mechanisms of carcinogenesis include chronic inflammation, proliferation of the bile duct epithelium, endogenous bile mutagens, and bile stasis. The majority of present clinical studies regarding CC selected PSC as a control, but PSC is rare in Eastern countries. In East Asia, particularly in Thailand, CC has been pathogenically associated with liver fluke infestation (Opisthorchis viverrini and Clonorchis sinensis) which increases the susceptibility of epithelial cell malignant transformation via chronic irritation and inflammation. In areas where Opisthorchis viverrini is endemic, the prevalence for CC when adjusted according to age and gender is as high as 14% [24], [25]. Given that the proposed mechanisms for CC formation involve chronic inflammation and bile stasis, choledocholithiasis and cholangitis are also considered as risk factors for CC which is uncommon in the West; in contrast, intra- and extrahepatic bile duct stones are much more common in Eastern Asia, including China [26]. Some studies have confirmed that hepatolithiasis is strongly associated with cholangiocarcinoma [1], [27], [28], and therefore we selected choledocholithiasis and cholangitis patients as the controls in the present study.

As mentioned previously, bile represents a proximal fluid that drains from the tumor microenvironment and therefore may contain an enriched source of potential serum biomarkers for early diagnosis [29]. In the present study, a classical 2D-PAGE proteomic approach was adopted to discover potential biomarkers of CC in human bile. As an extension of the proteomic research, the diagnostic value was validated by assessing the serum levels of one biomarker in CC using an ELISA.

Technically, a phase-nonionic-adsorbent and ultrafiltration protein purification method was adopted to pretreat the bile samples which enabled satisfactory resolution of 2-DE protein maps (Figure 1). High-abundance proteins were then depleted by columns containing immobilized antibodies against14 abundant plasma proteins, and an increased numbers of spots were observed in the 2-DE analysis, compared to previous reports [11], [12].

Several proteins previously associated with CC were identified in this study, including APOA1 [30], vimentin [31], [32] and PDIA3 [33]. For example, decreased APOA1 serum levels have been reported in patients with ovarian [34], pancreatic [35], and gastric cancer [36] as well as lymphoblastic leukemia [37]. SELDI-TOF-MS recently identified APOA1 as a potentially useful diagnostic biomarker for CC with a sensitivity of 80% and specificity of 76% [31].

Subsequent validation analyses of a series of individual bile samples confirmed the expression levels of selected candidate markers, to exclude any differences due to inter-individual variation (Figure 3). Moreover, these results demonstrated that the preliminary proteomic analysis generated reliable data for the discovery of novel and valuable candidate biomarkers for CC. We analyzed the distribution of PGAM1, HSPD1, PDIA3 and SSP411 in CC and adjacent normal tissues using immunoblotting and immunohistochemical staining, to confirm if these candidate biomarkers were derived from CC. Western blotting revealed that PGAM1, HSPD1, PDIA3 and SSP411 were expressed at high levels in CC compared to the matched normal tissues (Figure 4). Immunohistochemistry confirmed that SSP411 was upregulated in CC cells compared to match normal tissues. Additionally, intense expression of PGAM1, HSPD1, and PDIA3 was observed in the cytoplasm of cancer cells in both hilar (Figure 5) and intrahepatic CC (Figure S2). Simultaneously, the immune-cells around the tumor tissue also showed high immune-intensity for HSPD1 and PGAM-1. In contrast, SSP411 demonstrated more specific expression in the bile duct epithelium and in CC. For this reason SSP411 was selected for the subsequent ELISA analysis.

PGAM1 is a glycolysis enzyme which catalyzes interconversion of 3-phosphoglycerate and 2-phosphoglycerate with 2, 3-bisphosphoglycerate (2, 3-BPG) [38]. PGAM1 is overexpressed in breast cancer, and suppression of PGAM1 can inhibit breast cancer cell proliferation [39]. PGAM1 is also markedly upregulated in hepatocellular carcinoma (HCC) and has potential as a diagnostic biomarker and potential therapeutic target for HCC [40]. HSPD1 is typically localized in mitochondria and interacts with Hsp10 to chaperon nascent polypeptides. HSPD1 also interacts with Hsp70, survivin and p53 to participate in apoptosis. Recently, HSPD1 was associated with carcinogenesis, specifically tumor cell survival and proliferation, in different types of cancer [41], [42], [43]. This is the first report to suggest HSPD1 may be a potential biomarker of CC. ERp57 is a 58-kDa thiol oxidoreductase, detected in a variety of subcellular locations, and a member of the protein disulfide isomerase (PDI)-like family encoded by human *PDIA3*. The main function of ERp57 in the endoplasmic reticulum is quality control of newly synthesized glycoproteins, and assembly of major histocompatibility complex class 1 (MHC I). ERp57 is also involved in the modulation of STAT3 signaling-regulated gene expression and has been reported to be upregulated in other types of cancer [33], [44], [45]. SSP411 (also known as spermatogenesis-associated protein 20), a thioredoxin family member, is a novel spermatid-expressed gene which is thought to play a role in sperm maturation, fertilization and/or embryo development [46]. As previously mentioned, SSP411 is a testis-enriched gene which is not expressed in normal liver. This study provides the first evidence to suggest that SSP411 is overexpressed in bile from CC patients, suggesting that SSP411 may be a CC-associated biomarker. Promisingly, as a single biomarker, SSP411 could distinguish patients with CC from choledocholithiasis patients and normal individuals, suggesting that SSP411 may represent a potentially useful serum biomarker for the diagnosis of CC (Figure 6). Although the mean serum level of SSP411 in the benign group was higher than in the normal, there was no significant difference between the two groups. The ROC analysis also revealed that the serum level of SSP411 could not effectively differentiate benign disease from the normal individuals (Figure 6B). We speculated that this bias was attributed to the insufficient sample size, especially for the benign group. Similarly, no significant correlation was observed between the serum levels of SSP411 and lymph node metastasis or neural invasion in CC (data not shown), which may also be attributed to the small sample size of the negative patients. Further research is required to characterize the function of SSP411, which may also provide better understanding of the pathogenesis of CC.

In conclusion, this study demonstrates that 2-DE-based quantitative proteomic approaches are feasible for the discovery of disease biomarkers in bile. SSP411 represents a novel promising potential serum biomarker for CC. A study with a larger series of CC patients, including early stage patients, with a longer follow-up is currently in progress at our center to confirm the diagnostic accuracy and prognostic value of SSP411.

## Supporting Information

Figure S1 **BioGPS database analysis shows the tissue distribution of proteins identified by 2-DE.** (A) Protein was uniquely expressed in the human liver; (B) another protein was highly expressed in the liver or fetal liver; (C) SSP411 was a male germ cell-enriched gene.
(TIF)

Figure S2 **Immunohistochemical staining of PGAM-1, PDIA3, HSPD1 and SSP411 in intrahepatic cholangiocarcinoma (IHC) tissues.**
(TIF)

Table S1 **Identification of differentiated proteins using MALDI-TOF/TOF.**
(XLSX)

## References

[1] Tyson GL, El-Serag HB (2011) Risk factors for cholangiocarcinoma. Hepatology 54: 173–184. 21488076 10.1002/hep.24351 PMC3125451

[2] Blechacz BR, Gores GJ (2008) Cholangiocarcinoma. Clin Liver Dis 12: 131–150, ix. 18242501 10.1016/j.cld.2007.11.003

[3] Nehls O, Gregor M, Klump B (2004) Serum and bile markers for cholangiocarcinoma. Semin Liver Dis 24: 139–154. 15192787 10.1055/s-2004-828891

[4] Lee SG, Song GW, Hwang S, Ha TY, Moon DB, et al (2010) Surgical treatment of hilar cholangiocarcinoma in the new era: the Asan experience. J Hepatobiliary Pancreat Sci 17: 476–489. 19851704 10.1007/s00534-009-0204-5

[5] Hirano S, Kondo S, Tanaka E, Shichinohe T, Tsuchikawa T, et al (2010) Outcome of surgical treatment of hilar cholangiocarcinoma: a special reference to postoperative morbidity and mortality. J Hepatobiliary Pancreat Sci 17: 455–462. 19820891 10.1007/s00534-009-0208-1

[6] Nguyen K, Sing JT Jr (2008) Review of endoscopic techniques in the diagnosis and management of cholangiocarcinoma. World J Gastroenterol 14: 2995–2999. 18494049 10.3748/wjg.14.2995 PMC2712165

[7] Srinivas PR, Srivastava S, Hanash S, Wright GL Jr (2001) Proteomics in early detection of cancer. Clin Chem 47: 1901–1911. 11568117

[8] Kristiansen TZ, Bunkenborg J, Gronborg M, Molina H, Thuluvath PJ, et al (2004) A proteomic analysis of human bile. Mol Cell Proteomics 3: 715–728. 15084671 10.1074/mcp.M400015-MCP200

[9] Farina A, Dumonceau JM, Lescuyer P (2009) Proteomic analysis of human bile and potential applications for cancer diagnosis. Expert Rev Proteomics 6: 285–301. 19489700 10.1586/epr.09.12

[10] Farina A, Dumonceau JM, Frossard JL, Hadengue A, Hochstrasser DF, et al (2009) Proteomic analysis of human bile from malignant biliary stenosis induced by pancreatic cancer. J Proteome Res 8: 159–169. 19055478 10.1021/pr8004925

[11] Zhou H, Chen B, Li RX, Sheng QH, Li SJ, et al (2005) Large-scale identification of human biliary proteins from a cholesterol stone patient using a proteomic approach. Rapid Commun Mass Spectrom 19: 3569–3578. 16276486 10.1002/rcm.2207

[12] Chen B, Dong JQ, Chen YJ, Wang JM, Tian J, et al (2007) Two-dimensional electrophoresis for comparative proteomic analysis of human bile. Hepatobiliary Pancreat Dis Int 6: 402–406. 17690038

[13] Farina A, Dumonceau JM, Delhaye M, Frossard JL, Hadengue A, et al (2011) A step further in the analysis of human bile proteome. J Proteome Res 10: 2047–2063. 21314112 10.1021/pr200011b

[14] Farid SG, Craven RA, Peng J, Bonney GK, Perkins DN, et al (2011) Shotgun proteomics of human bile in hilar cholangiocarcinoma. Proteomics 11: 2134–2138. 21500345 10.1002/pmic.201000653

[15] Lankisch TO, Metzger J, Negm AA, Vosskuhl K, Schiffer E, et al (2011) Bile proteomic profiles differentiate cholangiocarcinoma from primary sclerosing cholangitis and choledocholithiasis. Hepatology 53: 875–884. 21374660 10.1002/hep.24103

[16] Huang XY, Guo XJ, Shen J, Wang YF, Chen L, et al (2008) Construction of a proteome profile and functional analysis of the proteins involved in the initiation of mouse spermatogenesis. J Proteome Res 7: 3435–3446. 18582094 10.1021/pr800179h

[17] Wu J, Tang Q, Shen J, Yao A, Wang F, et al (2010) Comparative proteome profile during the early period of small-for-size liver transplantation in rats revealed the protective role of Prdx5. J Hepatol 53: 73–83. 20451279 10.1016/j.jhep.2010.01.032

[18] Kokoszka JE, Waymire KG, Levy SE, Sligh JE, Cai J, et al (2004) The ADP/ATP translocator is not essential for the mitochondrial permeability transition pore. Nature 427: 461–465. 14749836 10.1038/nature02229 PMC3049806

[19] Agoston AT, Argani P, Yegnasubramanian S, De Marzo AM, Ansari-Lari MA, et al (2005) Increased protein stability causes DNA methyltransferase 1 dysregulation in breast cancer. J Biol Chem 280: 18302–18310. 15755728 10.1074/jbc.M501675200

[20] Shaib Y, El-Serag HB (2004) The epidemiology of cholangiocarcinoma. Semin Liver Dis 24: 115–25 8: 4092–4103. 15192785 10.1055/s-2004-828889

[21] Gatto M, Bragazzi MC, Semeraro R, Napoli C, Gentile R, et al (2010) Cholangiocarcinoma: update and future perspectives. Dig Liver Dis 42: 253–60. 20097142 10.1016/j.dld.2009.12.008

[22] Claessen MM, Vleggaar FP, Tytgat KM, Siersema PD, van Buuren HR (2009) High lifetime risk of cancer in primary sclerosing cholangitis. J Hepatol 50: 158–64. 19012991 10.1016/j.jhep.2008.08.013

[23] Burak K, Angulo P, Pasha TM, Egan K, Petz J, et al (2004) Incidence and risk factors for cholangiocarcinoma in primary sclerosing cholangitis. Am J Gastroenterol 99: 523–6. 15056096 10.1111/j.1572-0241.2004.04067.x

[24] Poomphakwaen K, Promthet S, Kamsa-Ard S, Vatanasapt P, Chaveepojnkamjorn W, et al (2009) Risk factors for cholangiocarcinoma in Khon Kaen, Thailand: a nested case-control study. Asian Pac J Cancer Prev 10: 251–8. 19537893

[25] Khan SA, Toledano MB, Taylor-Robinson SD (2008) Epidemiology, risk factors, and pathogenesis of cholangiocarcinoma. HPB (Oxford) 10: 77–82. 18773060 10.1080/13651820801992641 PMC2504381

[26] Okuda K, Nakanuma Y, Miyazaki M (2002) Cholangiocarcinoma: recent progress. Part 1: epidemiology and etiology. J Gastroenterol Hepatol 17: 1049–55. 12201863 10.1046/j.1440-1746.2002.02781.x

[27] Welzel TM, Graubard BI, El-Serag HB, Shaib YH, Hsing AW, et al (2007) Risk factors for intrahepatic and extrahepatic cholangiocarcinoma in the United States: a population-based case-control study. Clin Gastroenterol Hepatol 5: 1221–8. 17689296 10.1016/j.cgh.2007.05.020 PMC2083573

[28] Welzel TM, Mellemkjaer L, Gloria G, Sakoda LC, Hsing AW, et al (2007) Risk factors for intrahepatic cholangiocarcinoma in a low-risk population: a nationwide case-control study. Int J Cancer 120: 638–641. 17109384 10.1002/ijc.22283

[29] Koopmann J, Thuluvath PJ, Zahurak ML, Kristiansen TZ, Pandey A, et al (2004) Mac-2-binding protein is a diagnostic marker for biliary tract carcinoma. Cancer 101: 1609–1615. 15378479 10.1002/cncr.20469

[30] Wang X, Dai S, Zhang Z, Liu L, Wang J, et al (2009) Characterization of apolipoprotein A-I as a potential biomarker for cholangiocarcinoma. Eur J Cancer Care (Engl) 18: 625–635. 19486127 10.1111/j.1365-2354.2008.00965.x

[31] Korita PV, Wakai T, Ajioka Y, Inoue M, Takamura M, et al (2010) Aberrant expression of vimentin correlates with dedifferentiation and poor prognosis in patients with intrahepatic cholangiocarcinoma. Anticancer Res 30: 2279–2285. 20651380

[32] Dos Santos A, Court M, Thiers V, Sar S, Guettier C, et al (2010) Identification of cellular targets in human intrahepatic cholangiocarcinoma using laser microdissection and accurate mass and time tag proteomics. Mol Cell Proteomics 9: 1991–2004. 20513801 10.1074/mcp.M110.000026 PMC2938110

[33] Srisomsap C, Sawangareetrakul P, Subhasitanont P, Chokchaichamnankit D, Chiablaem K, et al (2010) Proteomic studies of cholangiocarcinoma and hepatocellular carcinoma cell secretomes. J Biomed Biotechnol 2010: 437143. 20069059 10.1155/2010/437143 PMC2801507

[34] Kozak KR, Su F, Whitelegge JP, Faull K, Reddy S, et al (2005) Characterization of serum biomarkers for detection of early stage ovarian cancer. Proteomics 5: 4589–4596. 16237736 10.1002/pmic.200500093

[35] Ehmann M, Felix K, Hartmann D, Schnolzer M, Nees M, et al (2007) Identification of potential markers for the detection of pancreatic cancer through comparative serum protein expression profiling. Pancreas 34: 205–214. 17312459 10.1097/01.mpa.0000250128.57026.b2

[36] Takaishi S, Wang TC (2007) Gene expression profiling in a mouse model of Helicobacter-induced gastric cancer. Cancer Sci 98: 284–293. 17270017 10.1111/j.1349-7006.2007.00392.x PMC11159662

[37] Halton JM, Nazir DJ, McQueen MJ, Barr RD (1998) Blood lipid profiles in children with acute lymphoblastic leukemia. Cancer 83: 379–384. 9669823

[38] de Atauri P, Repiso A, Oliva B, Vives-Corrons JL, Climent F, et al (2005) Characterization of the first described mutation of human red blood cell phosphoglycerate mutase. Biochim Biophys Acta 1740: 403–410. 15949708 10.1016/j.bbadis.2004.11.023

[39] Evans MJ, Saghatelian A, Sorensen EJ, Cravatt BF (2005) Target discovery in small-molecule cell-based screens by in situ proteome reactivity profiling. Nat Biotechnol 23: 1303–1307. 16200062 10.1038/nbt1149

[40] Ren F, Wu H, Lei Y, Zhang H, Liu R, et al (2010) Quantitative proteomics identification of phosphoglycerate mutase 1 as a novel therapeutic target in hepatocellular carcinoma. Mol Cancer 9: 81. 20403181 10.1186/1476-4598-9-81 PMC2873438

[41] Glaessgen A, Jonmarker S, Lindberg A, Nilsson B, Lewensohn R, et al (2008) Heat shock proteins 27, 60 and 70 as prognostic markers of prostate cancer. APMIS 116: 888–895. 19132982 10.1111/j.1600-0463.2008.01051.x

[42] Cappello F, David S, Rappa F, Bucchieri F, Marasa L, et al (2005) The expression of HSP60 and HSP10 in large bowel carcinomas with lymph node metastase. BMC Cancer 5: 139. 16253146 10.1186/1471-2407-5-139 PMC1289279

[43] Cappello F, Conway de Macario E, Marasa L, Zummo G, Macario AJ (2008) Hsp60 expression, new locations, functions and perspectives for cancer diagnosis and therapy. Cancer Biol Ther 7: 801–809. 18497565 10.4161/cbt.7.6.6281

[44] Cicchillitti L, Della Corte A, Di Michele M, Donati MB, Rotilio D, et al (2010) Characterisation of a multimeric protein complex associated with ERp57 within the nucleus in paclitaxel-sensitive and -resistant epithelial ovarian cancer cells: the involvement of specific conformational states of beta-actin. Int J Oncol 37: 445–454. 20596672 10.3892/ijo_00000693

[45] Turano C, Gaucci E, Grillo C, Chichiarelli S (2011) ERp57/GRP58: a protein with multiple functions. Cell Mol Biol Lett 16: 539–563. 21837552 10.2478/s11658-011-0022-z PMC6275603

[46] Shi HJ, Wu AZ, Santos M, Feng ZM, Huang L, et al (2004) Cloning and characterization of rat spermatid protein SSP411: a thioredoxin-like protein. J Androl 25: 479–493. 15223837 10.1002/j.1939-4640.2004.tb02819.x

