# Supplementary figures and images for: Comparative Proteomic Profiling of Human Bile Reveals SSP411 as a Novel Biomarker of Cholangiocarcinoma
Source: PLoS One. 2012 Oct 31;7(10):e47476. doi: 10.1371/journal.pone.0047476 (PMC3485295; doi:10.1371/journal.pone.0047476)

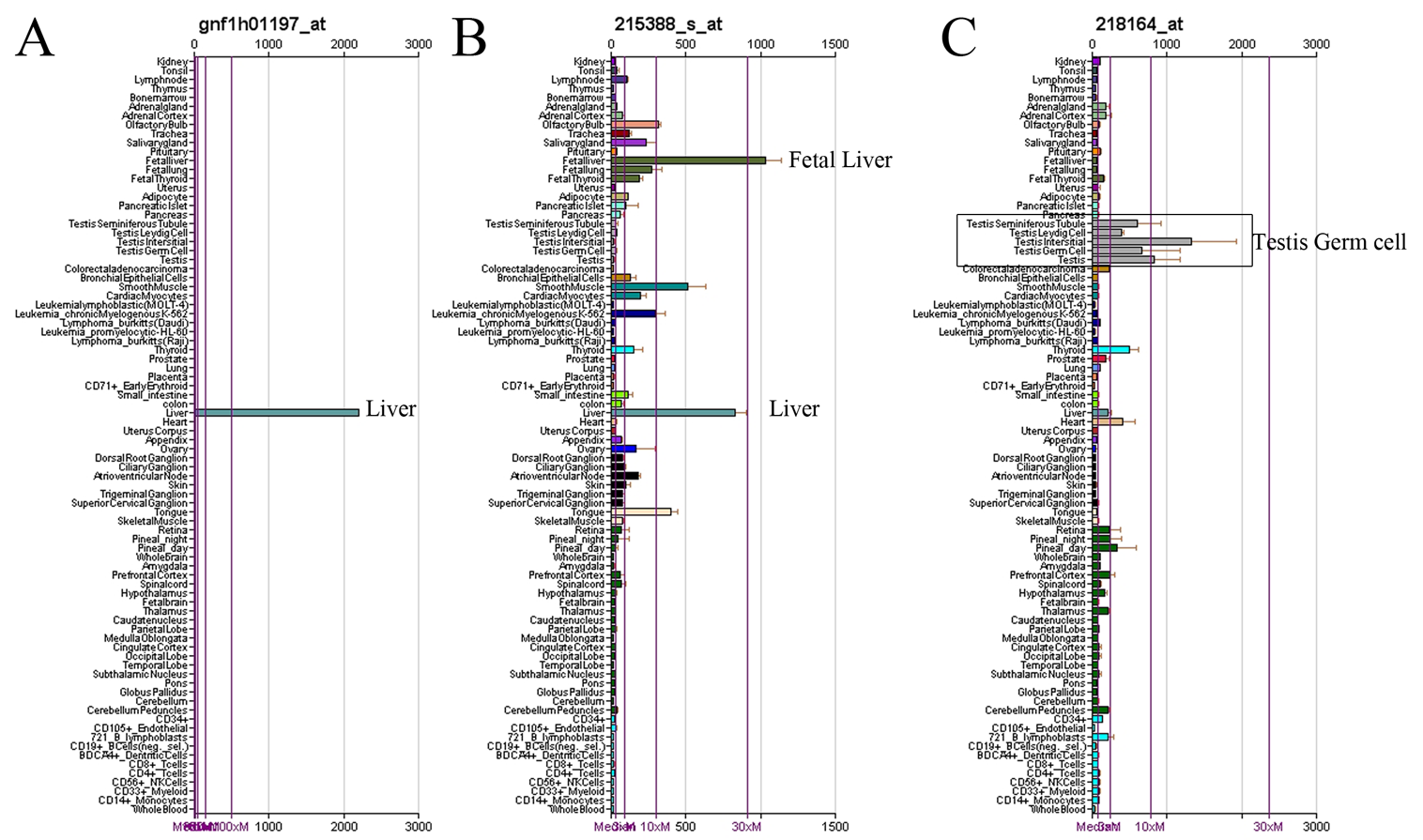

Supplement: Figure S1 — BioGPS database analysis shows the tissue distribution of proteins identified by 2-DE. (A) Protein was uniquely expressed in the human liver; (B) another protein was highly expressed in the liver or fetal liver; (C) SSP411 was a male germ cell-enriched gene. (TIF) [file pone.0047476.s001.tif]

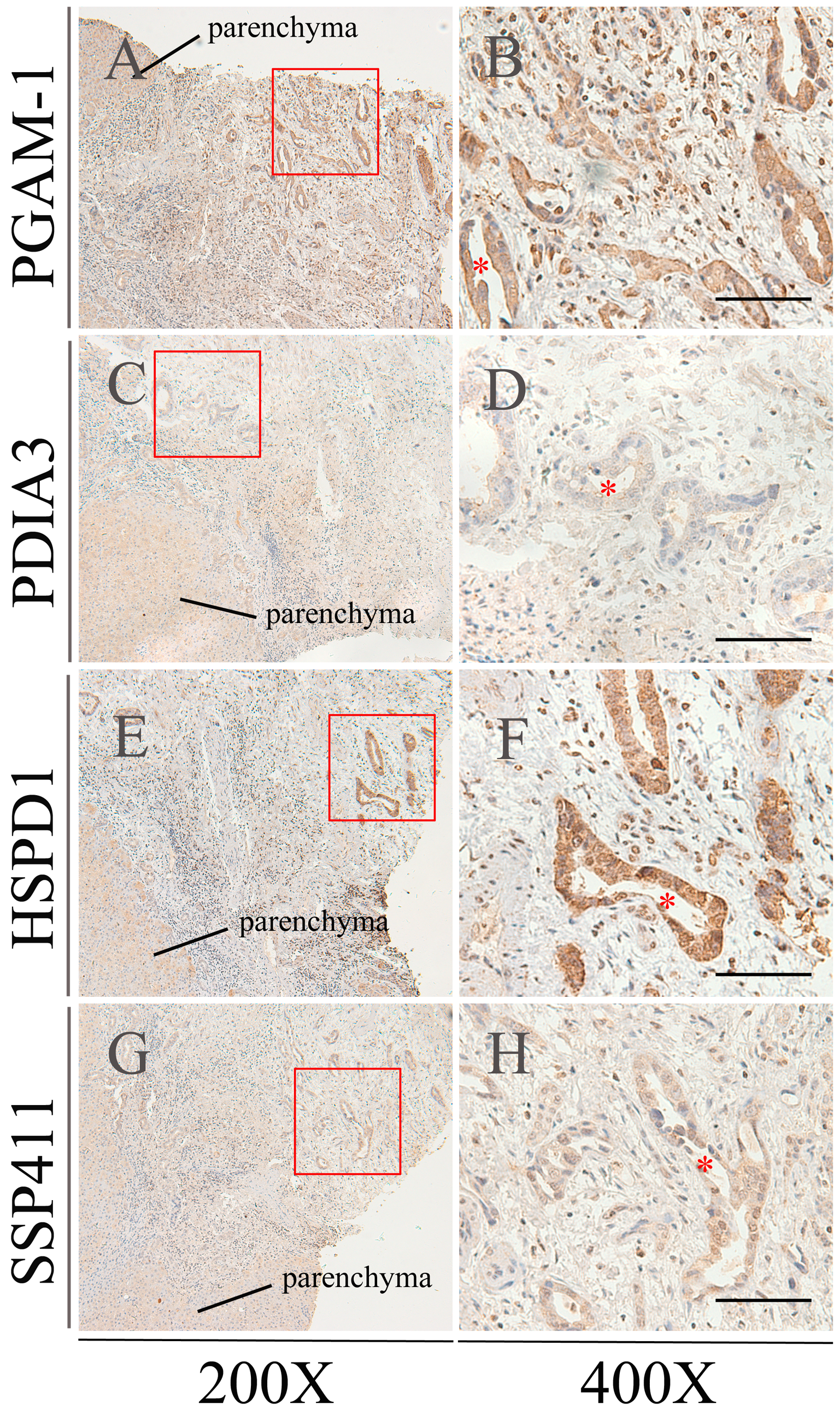

Supplement: Figure S2 — Immunohistochemical staining of PGAM-1, PDIA3, HSPD1 and SSP411 in intrahepatic cholangiocarcinoma (IHC) tissues. (TIF) [file pone.0047476.s002.tif]
